# Supplementary material for: MGP Promotes Colon Cancer Proliferation by Activating the NF-κB Pathway through Upregulation of the Calcium Signaling Pathway
Source: Mol Ther Oncolytics. 2020 Apr 19;17:371–83. doi: 10.1016/j.omto.2020.04.005 (PMC7210384; doi:10.1016/j.omto.2020.04.005)
Supplement: Document S1. Figures S1–S4 and Tables S1 and S3 [file mmc1.pdf]

OMTO, Volume *17*

Supplemental Information

MGP Promotes Colon Cancer Proliferation by Activating the NF-κB Pathway through Upregulation of the Calcium Signaling Pathway

Xueqing Li, Rui Wei, Mizhu Wang, Li Ma, Zheng Zhang, Lei Chen, Qingdong Guo, Shuilong Guo, Shengtao Zhu, Shutian Zhang, and Li Min

A

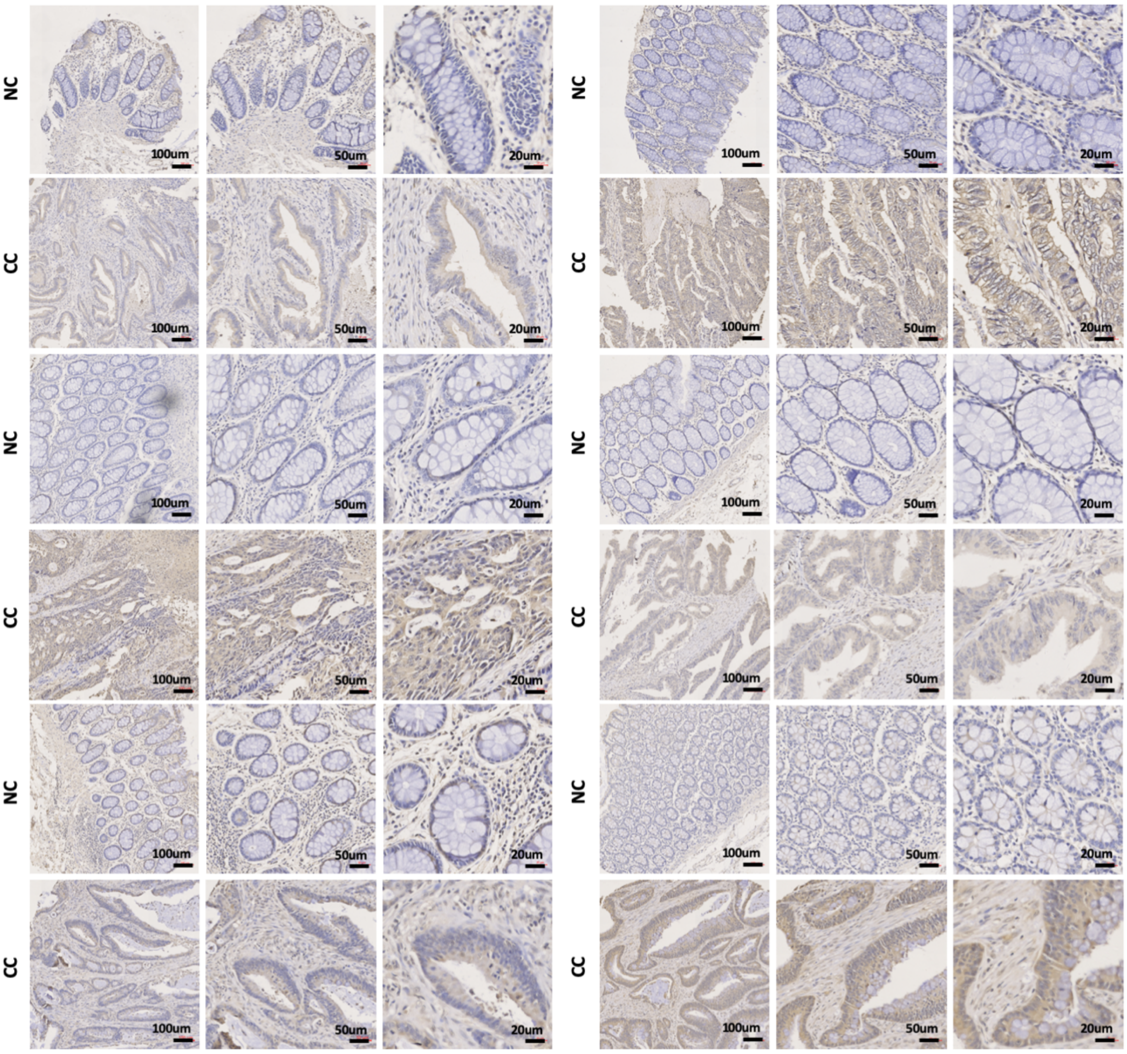

Figure S1. MGP is up-regulated in colon cancer patients.

(A) Representative IHC pictures of 80 pairs of CC patients. NC, adjacent non-tumor tissues; CC, colon cancer tissues.

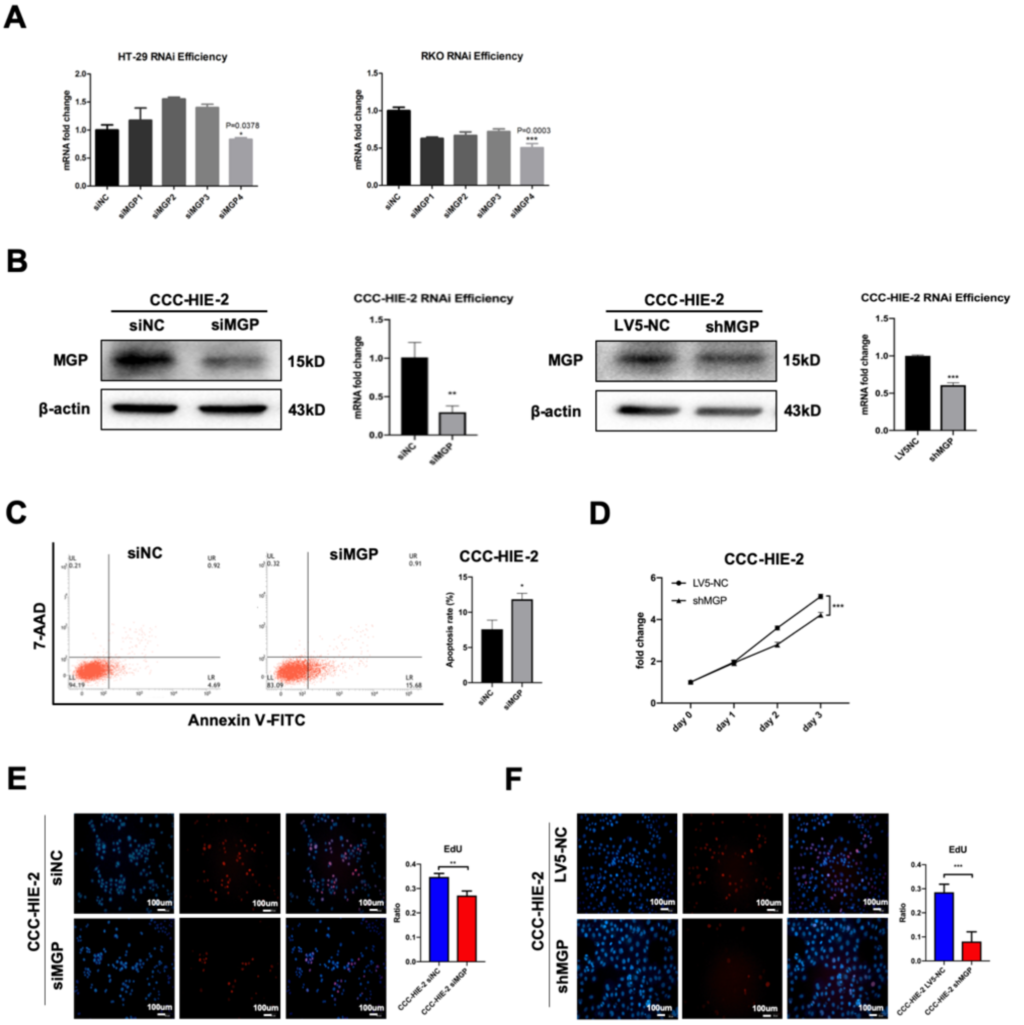

Figure S2. MGP inhibits cell apoptosis and promotes cell proliferation in normal colon epithelial cell.

(A) We designed four kinds of siRNA, and verified the knockdown efficiency by RT-qPCR after transfected with siRNAs in HT-29 and RKO cell lines. (B) The knockdown efficiency of normal colon epithelial cell line, CCC-HIE-2, was identified by western blotting and qRT-PCR. (C) The apoptosis ability of CCC-HIE-2 after transfected with siMGP. (D) The growth curve of CCC-HIE-2 after knockdown MGP. (E) EdU staining of CCC-HIE-2 transfected with siMGP. (F) EdU staining of CCC-HIE-2 stably transfected with LV5-NC and shMGP. Results are representative of 3 independent experiments. Values are the mean ±SD of the results. *p <0.05; **p <0.01; ***p <0.001.

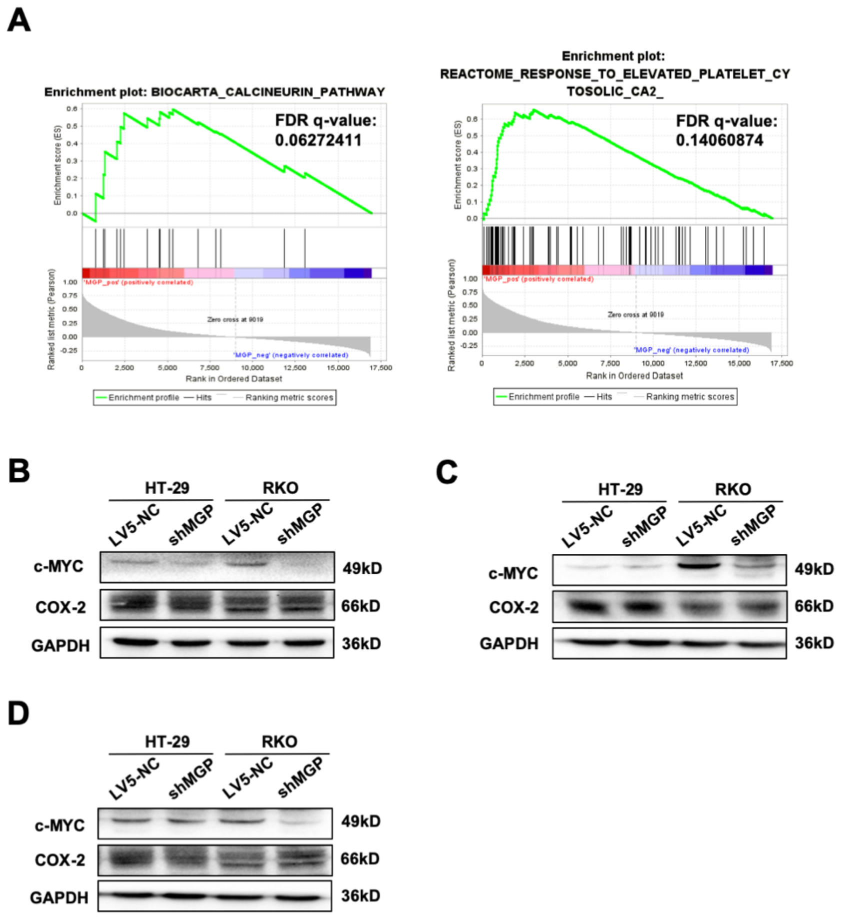

Figure S3. MGP is associated with calcium related pathways, and regulates NF-κB downstream genes expression in CC cells.

(A) GSEA analysis indicated that the expression level of MGP was correlated with up-regulated calcineurin pathway and elevated platelet cytosolic. (B, C, D) The protein level of NF-κB downstream genes when knockdown MGP.

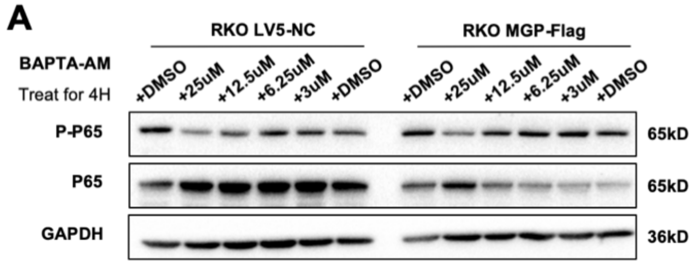

Figure S4. MGP regulating NF-κB pathway is blocked by BAPTA-AM in CC cells.

(A) Different concentrations of BAPTA-AM were cocultured with RKO cells for 4 hours. The protein level of p-NF-κB P65 was decreased at 25uM, with an increase expression of NF-κB P65.

Table S1. Clinical and pathological characteristics of 80 pairs of CC patients.

Variables	No. of patient
**Gender**	
Male	47 (58.75%)
Female	33 (41.25%)
**Age (years)**	
≥ 65	37 (46.25%)
< 65	43 (53.75%)
**T stage**	
T1, T2	15 (18.75%)
T3, T4	61 (76.25%)
*4 pairs of patients' information are not clear.	
**Lymph Node Metastasis**	
Negative	42 (52.50%)
Positive	38 (47.50%)
**Distant Metastases**	
Negative	69 (86.25%)
Positive	11 (13.75%)
**Pathological Stage**	
I-II, II	61 (76.25%)
II-III, III	19 (23.75%)
**Clinical Stage**	
I, II	39 (48.75%)
III, IV	41 (51.25%)

Table S2. The detailed histology scores of 80 pairs of colon cancer patients' tissues. (Excel)

Table S3. Information of primary antibodies in WB and IF.

Primary Antibody	Company	Cat.	Dilution Factor
β-actin	Abbkine	A01010	WB 1:5000
MGP	Santa	SC-81546	IHC 1:100
			WB 1:500
			IF 1:50
NF-κB P65	Proteintech	10745-1-AP	WB 1:2000
			IF 1:50
phospho-NF-κB P65	HuaBio	ET1604-27	WB 1:1000
			IF 1:50
CREB	CST	9197	WB 1:1000
phospho-CREB	CST	9198	WB 1:1000
NFATC1	Abcam	Ab177464	WB 1:1000
phospho-NFATC1	R&D	679340	WB 1:1000
c-MYC	Proteintech	10828-1-AP	WB 1:2000
COX-2	Proteintech	12375-1-AP	WB 1:2000
GAPDH	Abbkine	A01020	WB 1:5000
Lamin B1	CST	9087S	WB 1:1000

Table S4. Details and the raw data of the GSEA (KEGG) analysis for MGP. (Excel)

Table S5. Details and the raw data of the GSEA (BIOCARTA) analysis for MGP. (Excel)
