# Supplementary material for: MGP Promotes Colon Cancer Proliferation by Activating the NF-κB Pathway through Upregulation of the Calcium Signaling Pathway
Source: Mol Ther Oncolytics. 2020 Apr 19;17:371–83. doi: 10.1016/j.omto.2020.04.005 (PMC7210384; doi:10.1016/j.omto.2020.04.005)
Supplement: Document S2. Article plus Supplemental Information [file mmc5.pdf]

Molecular Therapy
Oncolytics
Original Article

MGP Promotes Colon Cancer Proliferation by Activating the NF-κB Pathway through Upregulation of the Calcium Signaling Pathway

Xueqing Li,[1,2,3] Rui Wei,[1,3] Mizhu Wang,[1] Li Ma,[2] Zheng Zhang,[1] Lei Chen,[1] Qingdong Guo,[1] Shuilong Guo,[1] Shengtao Zhu,[1] Shutian Zhang,[1] and Li Min[1]

[1]Department of Gastroenterology, Beijing Friendship Hospital, Capital Medical University, National Clinical Research Center for Digestive Disease, Beijing Digestive Disease Center, Beijing Key Laboratory for Precancerous Lesion of Digestive Disease, Beijing 100050, P.R. China; [2]Department of Gastroenterology, The Second Hospital of Shanxi Medical University, Taiyuan, Shanxi 030001, P.R. China

Matrix Gla protein (MGP), an extracellular matrix protein, is mainly associated with the inhibition of calcification in skeleton, coronary artery, and kidney, and more recently it has also been implicated in cancer. However, the biological function of MGP inside cancer cells and its role in colon cancer (CC) remain largely unknown. MGP expression and its association with clinicopathologic characteristics in CC were analyzed by immunohistochemistry and verified by Gene Expression Omnibus (GEO) and The Cancer Genome Atlas (TCGA) datasets. The effects of MGP on CC cell proliferation were evaluated via knockdown and overexpression experiments *in vitro*. Mechanisms of MGP in CC were explored by western blots, quantitative real-time PCR, Fluo-3 AM staining, Rhod-2 AM staining, immunofluorescence, and other techniques. Our study confirmed that MGP was upregulated in different stages of CC and associated with a worse prognosis. MGP could enrich intracellular free Ca^{2+} concentration and promote nuclear factor κB (NF-κB)/p65 phosphorylation, activating the expression of c-MYC, ICAM-1, and VEGFA. Furthermore, the reduction of intracellular free Ca^{2+} concentration and the subsequent growth inhibition effect on CC cells induced by small interfering RNA targeting MGP (siMGP) could be rescued by a higher calcium concentration environment. Therefore, MGP promotes the growth and proliferation of CC cells by enriching intracellular calcium concentration and activating the NF-κB pathway, and it could serve as a potential prognostic biomarker in CC patients.

INTRODUCTION

Colon cancer (CC) makes up the second largest contribution to cancer-related mortality worldwide, accounting for around 1.8 million new patients and 881,000 deaths in 2018.[1] Despite considerable improvements in surgery, as well as several combination chemotherapy regimens with cytotoxic drugs and molecular-targeted agents, the 5-year survival rate remains low in advanced CC patients.[2,3] Therefore, the discovery of new biomarkers and the identification of new drug targets for CC are of vital importance.[4,5]

Matrix Gla protein (MGP) is a secreted, calcium-binding matrix protein that contains five to six post-translationally modified γ-carboxyglutamic acid residues originating from vitamin K-dependent carboxylation.[6,7] MGP is synthesized in cartilage, bone, and other tissues, such as lung, heart, kidney, and vascular smooth muscle cells.[6,8,9] Initially, MGP plays a role as a physiological inhibitor of ectopic tissue calcification (e.g., cartilaginous and vascular tissues), pathological calcifications (e.g., osteoarthritis), and microcalcification (in atherosclerotic coronary arteries).[10,11] Interestingly, mutations in this gene coding for MGP can cause Keutel syndrome (KS) in human patients, leading to ectopic abnormal calcification and midfacial hypoplasia, which substantiates the role of MGP in extracellular matrix (ECM) calcification regulation.[12,13] There have been many studies on the function of MGP in the ECM,[14] but the exact biological functions of intracellular MGP are not clear.

It has been known that MGP binds calcium ions (Ca^{2+}) through γ-carboxylated glutamates and renders conformational changes in the MGP protein, confirmed by analytical high-pressure liquid chromatography (HPLC).[15,16] Warfarin interferes with the vitamin K-dependent Gla residues through loss of calcium-binding and renders MGP nonfunctional. MGP is found in high concentrations in bone and cartilage.[6,17] It was reported that MGP functioned as a bone morphogenetic protein (BMP) inhibitor, and the deficiencies of MGP caused impaired osteogenic differentiation and calcification.[18] BMP-binding and calcium-binding functions of MGP have been confirmed as two

Received 27 February 2020; accepted 14 April 2020;
https://doi.org/10.1016/j.omto.2020.04.005.

[3]These authors contributed equally to this work.

Correspondence: Li Min, PhD, Department of Gastroenterology, Beijing Friendship Hospital, Capital Medical University, National Clinical Research Center for Digestive Disease, Beijing Digestive Disease Center, Beijing Key Laboratory for Precancerous Lesion of Digestive Disease, Beijing 100050, P.R. China.
E-mail: minli@ccmu.edu.cn

Correspondence: Shutian Zhang, PhD, Department of Gastroenterology, Beijing Friendship Hospital, Capital Medical University, National Clinical Research Center for Digestive Disease, Beijing Digestive Disease Center, Beijing Key Laboratory for Precancerous Lesion of Digestive Disease, Beijing 100050, P.R. China.
E-mail: zhangshutian@ccmu.edu.cn

This is an open access article under the CC BY license (http://creativecommons.org/licenses/by/4.0/).

intertwined processes, and they are essential for the prevention of vascular calcification.[19]

Ca^{2+} is important for cellular signaling. When entering the cytoplasm, Ca^{2+} exerts regulatory effects on many enzymes and proteins by the coordinated activity of calcium channels, pumps, exchangers, and binding proteins.[20,21] Recently, the communication between Ca^{2+} and tumor cells and how Ca^{2+} influences cancer cell growth and cycling functions were largely revealed.[22] It has been reported that an excessive concentration of Ca^{2+} could induce tumor cell apoptosis by regulating molecular mechanisms in the nucleus.[23,24] Moreover, Ca^{2+} has also been explored as the key factor in mediating lymphocyte development and function initiated by stromal interaction molecule (STIM) /Orai-dependent Ca^{2+} signals, including the nuclear factor of activated T cells (NFAT) and nuclear factor κB (NF-κB) pathways.[25,26]

In this study, we investigated the role of MGP in CC and revealed that its overexpression was associated with an unfavorable prognosis. We also showed a pro-proliferation effect of MGP inside CC cells, by up-regulating intracellular free Ca^{2+} concentration and thus targeting NF-κB-dependent gene expressions.

RESULTS

MGP Is Overexpressed in CC and Indicates a Worse Clinical Prognosis

To explore the expression pattern of MGP in CC, we performed immunohistochemistry (IHC) assays in 80 pairs tissues of CC patients and their matched normal tissues. (The clinical and pathological characteristics of patients are listed in Table S1. The detailed histology scores of 80 pairs of tissues are listed in Table S2.) The results suggested that MGP primarily localized to the luminal epithelium and was expressed in the cytoplasm and partially in the nucleus (Figure 1A). Additionally, in some advanced CC patients, MGP was also expressed in the ECM and other parts of the tumor microenvironment. More representative images are also provided in Figure S1A. MGP in CC cells exhibited a significantly higher expression compared with adjacent non-tumor tissues by IHC ($p < 0.001$, Figure 1B). MGP level was associated with advanced tumor stage. As compared to normal colon tissues, the IHC score of CC patients increased along with the increase of clinical stage (Figure 1C). Additionally, MGP expression was also positively correlated with pathological classification (Figure 1D). Analysis of data from two independent Gene Expression Omnibus (GEO) colorectal cancer (CRC) datasets (GEO: GSE6988 and GSE20842) suggested that MGP was overexpressed in CC (Figure 1E) and rectal cancer (RC) (Figure 1F) at the mRNA level. Additionally, analysis of data from The Cancer Genome Atlas (TCGA) CRC patients suggested that higher MGP expression was correlated with worse overall survival ($\chi^2 = 8.7$, $p = 0.0032$, Figure 1G) and disease-free survival ($\chi^2 = 6.6$, $p = 0.0103$, Figure 1H).

MGP Enhances Cell Proliferation and Inhibits Cell Apoptosis in CC

To reveal the role of MGP in colon carcinogenesis, we blocked the expression of MGP in HT-29 and RKO cell lines. We chose the most potent small interfering RNA (siRNA) targeting MGP (siMGP) of four siRNAs by quantitative real-time PCR (Figure S2A). Knockdown efficiency was confirmed by western blot (Figure 2A). Remarkably, MTS (3-(4,5-dimethylthiazol-2-yl)-5-(3-carboxymethoxyphenyl)-2-(4-sulfophenyl)-2*H*-tetrazolium) assays indicated that the cell viability of MGP knockdown cells was decreased in both HT-29 and RKO cells (Figure 2B). In addition, the reduction of MGP led to a significantly increase in early-stage cell apoptosis (Figure 2C). As for the colony-forming assay, the results showed that MGP knockdown largely inhibited the colony formation ability of those two CC cell lines (Figures 2D and 2E). We also overexpressed the MGP expression by a lentivirus vector, and altered expression of MGP was confirmed by western blot (Figure 2F). In addition, as supported by the results of MTS assays (Figure 2G), we concluded that higher expression of MGP could promote CC cell proliferation. EdU (5-ethynyl-2′-deoxyuridine) staining was also performed, which suggested that MGP could promote cell division and proliferation after MGP was overexpressed in HT-29 and RKO cells (Figure 2H). In the normal colon epithelial cell line CCC-HIE-2, knockdown of MGP by RNA interference or RNA-short hairpin RNAs by lentivirus led to inhibiting cell growth through reducing proliferation (Figures S2D–S2F) and also increasing cell apoptosis (Figure S2C).

MGP Promotes Progression of CC via the Ca^{2+}-NF-κB Signaling Pathway

To elucidate the potential mechanism underlying the pro-proliferation effect of MGP, gene set enrichment analysis (GSEA) was performed and revealed that the expression level of MGP was correlated with the calcium signaling pathway (Figure 3A). It was reported that MGP could bind Ca^{2+} through Gla residues and effectively prevented vascular calcification.[15] Elevated phosphate and Ca^{2+} levels were also found to induce an increase of MGP protein and gene expression.[27] To test how MGP affected Ca^{2+} in CC cytoplasm, we performed Fluo-3 AM staining in HT-29 and RKO cells transfected with MGP siRNA and normal control siRNA (siNC), and showed a decrease of Ca^{2+} concentration in cytoplasm and nucleus of siMGP cells (Figures 3B–3D). Then, we collected RKO cells and used fluorescence-activated cell sorting (FACS) software to compare the live cell count numbers at a certain fluorescence intensity. As shown in Figure 3E, the red line showed a left shift as compared to the purple line, which suggested that siMGP decreased the intracellular free Ca^{2+} fluorescence intensity of RKO cells as compared to siNC cells. The above results demonstrated that Ca^{2+} calcium concentration is distinctly reduced after MGP is knocked down.

It has been reported that Ca^{2+} activates NF-κB, NFAT, and cyclic AMP response element binding protein (CREB) pathways.[25,28] In this study, we revealed that the expression level of MGP was correlated with NF-κB, CREB, and NFAT signaling pathways in GSEA (Figure 4A). Also, MGP expression was found correlated with the calcineurin pathway and response to elevated platelet cytosolic Ca^{2+} (Figure S3A). All details and the raw data of the GSEA about MGP are displayed in Tables S4 and S5. To reveal the underlying oncogenic mechanism of MGP, we also detected proteins associated with

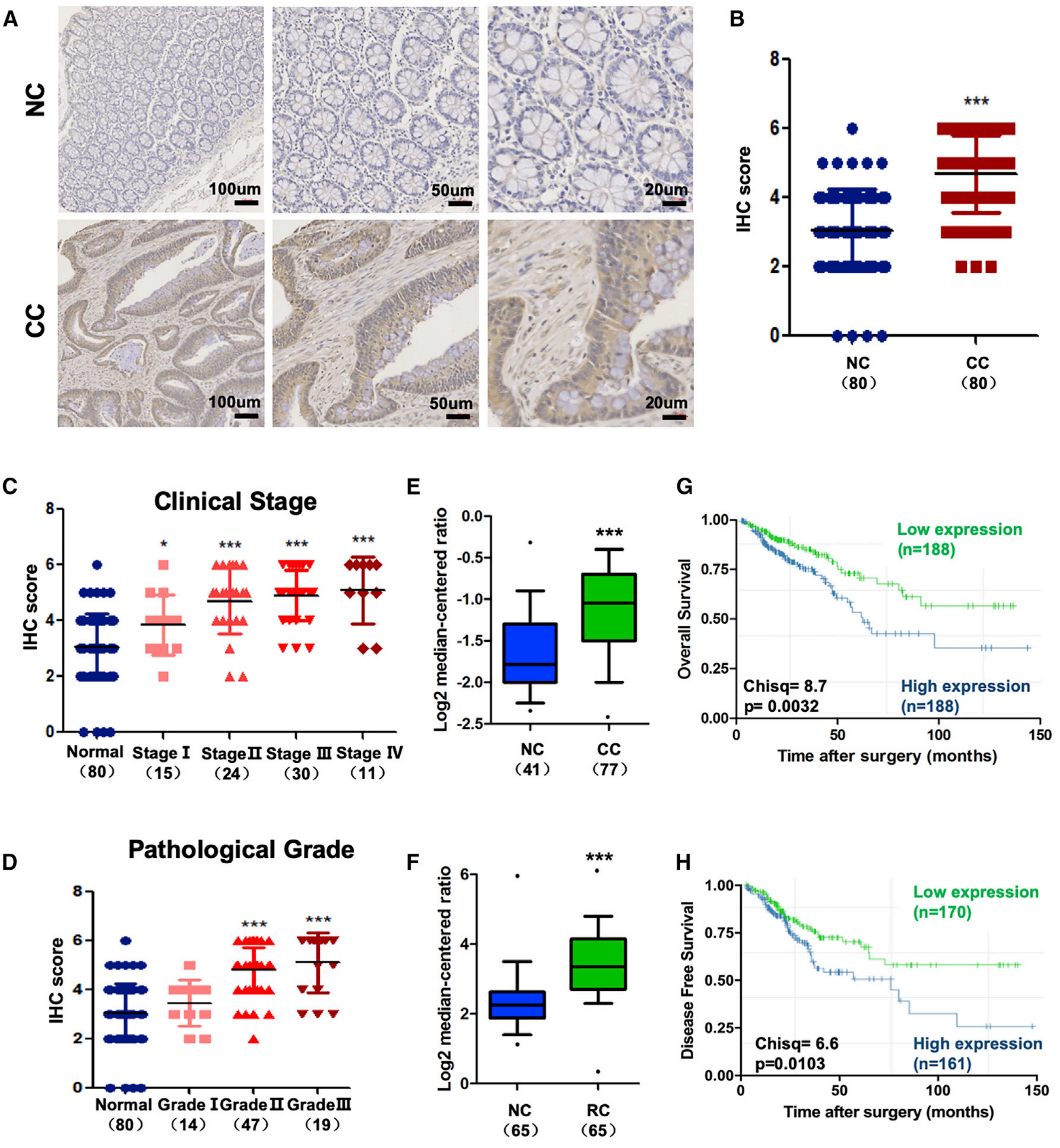

Figure 1. MGP Is Overexpressed in Colon Cancer and Is Associated with Poor Prognosis

(A) Representative images of MGP measured by IHC in CC and adjacent normal tissues. NC, adjacent non-tumor tissues; CC, colon cancer tissues. (B) IHC staining scores for 80 pairs of CC and adjacent normal tissues. Data are shown as mean ± SD. (C and D) IHC staining scores of four different American Joint Committee on Cancer (AJCC) stages (C, clinical stage; D, pathological grade). CC cells were analyzed and compared with normal colon tissues. Data are shown as mean ± SD. (E and F) MGP expression differences between normal colon tissues and CRC in mRNA level based on the NCBI Gene Expression Omnibus (GEO: GSE6988 and GSE20842). Data are shown in the form of boxplots (E, NC and CC tissues; F, NC and RC tissues). The OS (G) and DFS (H) of CC patients stratified by MGP expression levels are shown. *$p < 0.05$, ***$p < 0.001$.

NF-κB/p65 pathway activation. Our results indicated that in both HT-29 and RKO cell lines, siRNA silencing of MGP resulted in decreased phosphorylation level of NF-κB/p65, CREB, and NFATC1, whereas the total protein levels of NF-κB/p65, CREB, and NFATC1 remained unchanged (Figure 4B, left panel). NF-κB signaling, one of the most important pathways in carcinogenesis, was selected for further investigation. As shown in Figure 4B (right panel), siRNA silencing of MGP resulted in a decrease of NF-κB-promoted genes, such as c-MYC and COX-2. Three independent experiments of western blotting are provided in Figure S3B. Collectively, our finding indicated that MGP could promote NF-κB phosphorylation and regulate its downstream gene expression.

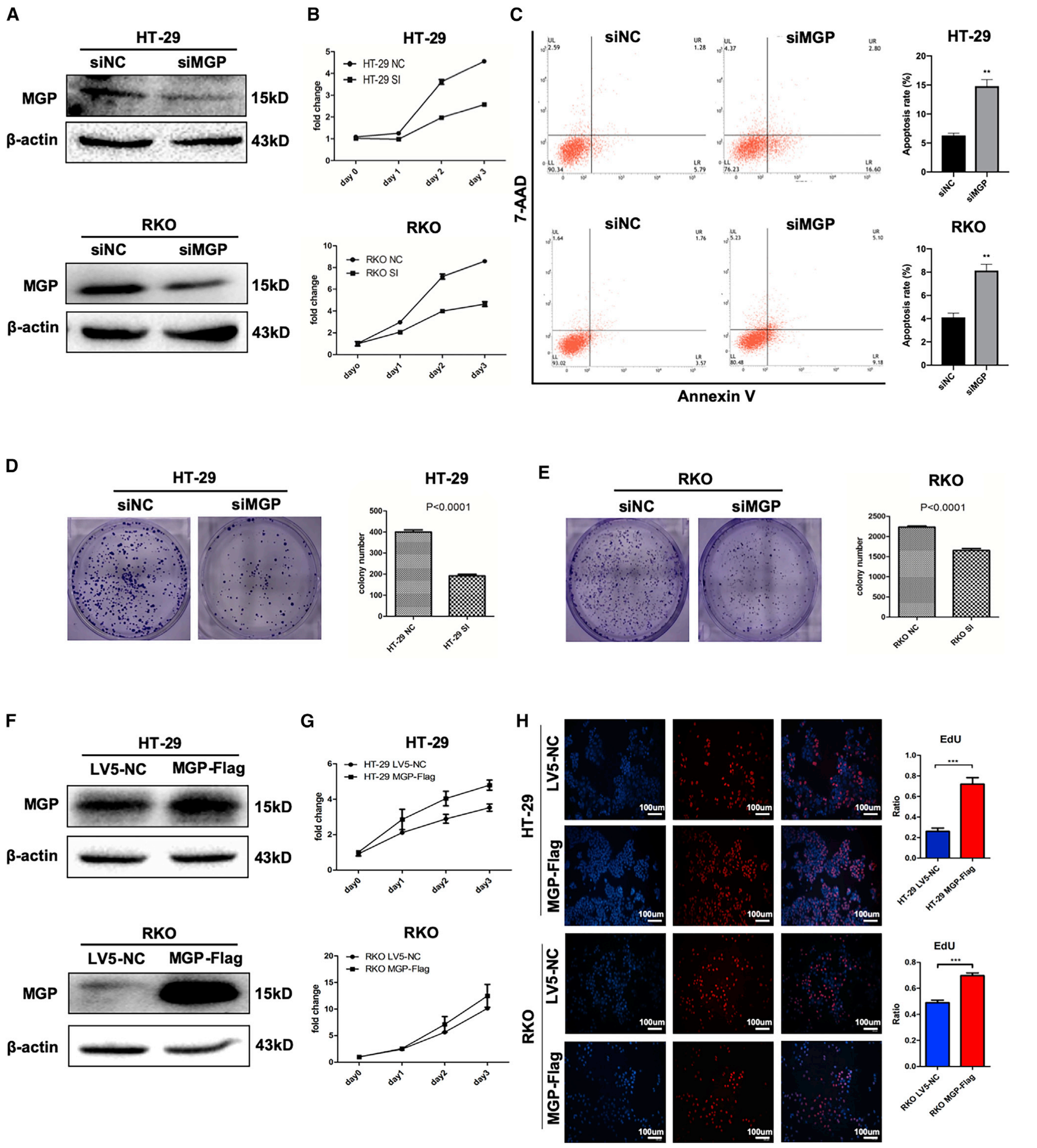

Figure 2. MGP Promotes Cells Growth and Proliferation in CC Cell Lines

(A) MGP protein expression levels were knocked down by siRNA in HT-29 and RKO cell lines. (B) Growth curves of HT-29 and RKO cells treated with scrambled siRNA (siNC) or MGP siRNA. (C) Apoptosis ability of HT-29 and RKO cells was evaluated after knocking down the expression of MGP. (D and E) Colony formation assay of HT-29 (D) and

(legend continued on next page)

MGP Participates in the Regulation of NF-κB Downstream Genes

Our previous results indicated that MGP activated NF-κB p65 through upregulating intracellular Ca^{2+}. To confirm this phenomenon, we performed a nucleus and cytoplasm protein separation/extraction assay and found that the protein level of phosphorylated (p-)p65 in nucleus was largely reduced by siMGP (Figure 5A). Furthermore, in immunofluorescence assays, we also confirmed that p-p65 in the nucleus was significantly decreased under MGP siRNA treatment in both HT-29 and RKO cell lines (Figure 5B).

We extracted and analyzed the data from TCGA and identified strongly positive correlations between MGP and the NF-κB downstream molecules (Figure 5C). According to datasets from TCGA CRC database, we predicted the expression of MGP at the mRNA level was positively correlated with MCL1 ($p = 0.005$), BCL2 ($p < 0.001$), ICAM-1 ($p < 0.001$), and ADH1B ($p = 0.003$). After transfecting HT-29 and RKO cells with siMGP, quantitative real-time PCR was applied to evaluate the mRNA fold changes of those genes. Our results suggested that putative target genes such as MCL1, ICAM-1, and VEGFA were significantly downregulated when knocking down MGP in both the HT-29 and RKO cell lines (Figure 5D). In overexpressed MGP cell lines, ICAM-1 and VEGFA were increased in both CC cell lines (Figure 5E).

Growth Inhibition Resulting from siMGP Could Be Rescued by Increasing the Ca^{2+} Concentration in CC Cells

In order to identify whether MGP promotes CC proliferation in a Ca^{2+}-dependent manner, rescue assays were performed with direct addition of calcium ion reagent. After a 6-h transfection of CC cells, two groups were replaced with DMEM complete media containing 0.1 or 0.3 mg/mL calcium ion. We found that the intracellular fluorescence intensity of CC cells was largely increased in the higher calcium concentration environment (Figures 6A and 6B), suggesting that Ca^{2+} was well absorbed by the CC cells. Additionally, we found that the colony-forming and proliferation abilities were partially reversed in MGP-blocked cells (Figures 6C–6E). The levels of p-p65 protein were upregulated with increasing intracellular Ca^{2+} concentrations in HT-29 and RKO cell lines as well (Figure 6F).

Increased Growth Resulting from Overexpressing MGP Could Be Blocked by Sequestering Ca^{2+} Ions in CC Cells

To test the possibility that the exerting pro-proliferation ability of MGP was mediated by the calcium signaling pathway, CC cells that were stably transfected with lentivirus vector (MGP-Flag) were treated with BAPTA-AM (1,2-bis(2-aminophenoxy)ethane-*N*,*N*,*N*′,*N*′-tetraacetic acid acetoxymethyl ester) (an intracellular Ca^{2+} chelator, dissolved in DMSO). We used different drug concentrations treating CC cells and finally found that under the concentration of 25 μM for 4 h, the intracellular Ca^{2+} was significantly chelated (Figures 7A and 7B). Then, the effects of diverse concentrations of BAPTA-AM on proliferation ability in CC cells were displayed (Figures 7C and 7D). With the growing concentration of BAPTA-AM, the inhibition on cell growth was more significant. In addition, the effects of different BAPTA-AM concentrations on the protein level of p-p65 were shown and suggested that 25 μM could be the effective drug concentration (Figure S4A). Collectively, the protein level of p-p65 was downregulated with decreasing intracellular free Ca^{2+} concentrations in HT-29 and RKO cell lines (Figures 7E and 7F).

DISCUSSION

Recent studies have shown that overexpressed MGP could be found in various types of cancer, including breast cancer, glioblastoma, primary renal cancer, testicular cancer, prostatic carcinomas, and ovarian cancer, and they suggested that it may be associated with tumor progression and invasion.[29] In this study we revealed that MGP expression was increased in CC tissue based on IHC staining. The overexpression of MGP was also verified by GEO CRC datasets, which made our conclusion more credible. In summary, we identified MGP as a prognostic factor of CC patients, which further highlighted the potential for MGP to serve as a valuable marker. Also, we noted that Fan et al.[30] used 80 pairs of colorectal adenocarcinoma tissues to show that the mRNA level of MGP was reduced in CRC (79%) compared to adjacent normal tissues by northern blot. The inconsistency between our results and those of Fan et al.[30] could be mainly caused by different sample composition (70/80 cases in Fan et al.'s study were left-sided CRC) and tumor heterogeneity. Additionally, northern blot only detects a certain region of MGP mRNA, whereas sequencing gives an unbiased description of all full-length MGP transcripts. Considering the consistency between the TCGA sequencing data and our IHC results, we tend to think that MGP is generally overexpressed in CC as compared to normal colon tissues, but we do not completely rule out the possible low MGP expression in certain CC cases.

As we have shown in the present study, this gene was found mainly as a secreted protein and localized in the ECM of vascular smooth muscle cells, cartilage, bone, and heart. However, we found in CC tissues that the expression of MGP was mainly distributed among the luminal epithelium, so that the possible intracellular function of MGP was largely neglected. We suggested that this phenomenon was associated with the complicated secretory mechanism of MGP in cancer cells. Meanwhile, we found that MGP was also expressed in the ECM and other parts of the tumor microenvironment, such as fibroblasts and immune cells. Ostman et al.[31] revealed that MGP was upregulated in fibroblasts of human basal cell carcinoma at mRNA level; however, the authors found that the mRNA expression level of MGP in fibroblasts of CC was downregulated, compared to

RKO (E) cell lines. (F) MGP protein expression levels were overexpressed by MGP-Flag in HT-29 and RKO cell lines. (G) Growth curves of HT-29 and RKO cells treated with LV5-NC and MGP-Flag. (H) EdU staining of HT-29 and RKO cells stably transfected with LV5-NC and MGP-Flag. Results are representative of three independent experiments. Values are the mean ± SD of the results. $^{**}p < 0.01$, $^{***}p < 0.001$.

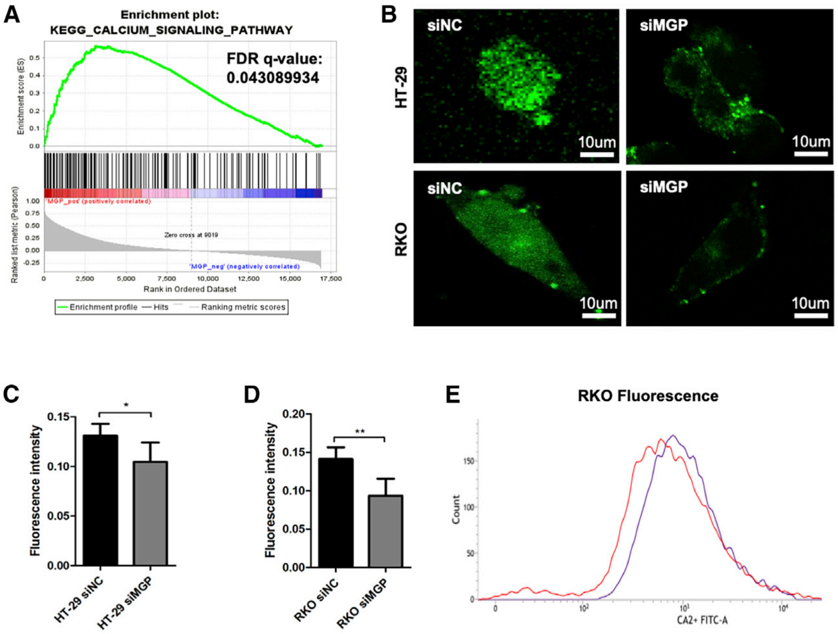

Figure 3. MGP Increases Ca^{2+} Concentration in Cytoplasm of CC Cells

(A) GSEA indicated that the expression level of MGP was correlated with the calcium signaling pathway. (B) Fluo-3 AM staining (green) of HT-29 and RKO cell lines transfected with scrambled siRNA and MGP siRNA. (C and D) Data for the mean relative fluorescence intensity (MRFI) of (B) (C, HT-29 cells; D, RKO cells). (E) We used FACS software for comparing the fluorescence intensity of intracellular calcium after incubation with Fluo3-AM. The density plot represents the intracellular free calcium concentration of RKO cells after transfection with siNC (purple line) and siMGP (red line). Results are representative of three independent experiments. Values are the mean ± SD of the results. $^{*}p < 0.05$, $^{**}p < 0.01$.

normal fibroblasts of normal tissue. The difference in MGP abnormal expression of different cancers could be a result of tumor heterogeneity. Thus, the expression and function of MGP in the tumor microenvironment needs further investigation.

Maintaining the homeostasis of Ca^{2+} concentrations both intracellularly and extracellularly is essential for cellular function, ranging from active secretion and movement to cell differentiation and death. Numerous studies have shown that four major Ca^{2+} influx pathways, localized in plasma membrane, are involved in the regulation of $[Ca^{2+}]_i$ (intracellular Ca^{2+} concentration) homeostasis, including voltage-gated Ca^{2+} channels (e.g., L-type), permeable Ca^{2+} channels mediated by the transient receptor potential (TYP) family, purinergic receptors (e.g., P2X), and the SOCE (store-operated calcium entry) pathway mediated by the components of ORAI1 and STIM1. In addition, Ca^{2+} could also be released from intracellular stores such as endoplasmic reticulum (ER) and Golgi apparatus (GA) into the cytosol.[32] When there were changes in cytosolic free Ca^{2+} concentration, cells could finely maintain the balance by regulating Ca^{2+} influx and efflux.[33] Increasing evidence has suggested that there are differences in regulating Ca^{2+} signaling between cancer cells and other cells, such as normal cells and vascular smooth muscle injury cells, among others.[22] It recently became the consensus that cancer cells modify the mitochondria-associated Ca^{2+} channels, which may affect calcium homeostasis to support epithelial-mesenchymal transdifferentiation and resistance to apoptosis.[34] In addition, it was also reported that the Ca^{2+} protein alpha-1D of the CaV1.3 channel was overexpressed in CC cells and was responsible for regulating migration ability and Ca^{2+} homeostasis.[35] However, the working mechanisms of Ca^{2+} channels are complicated, and their relevance to tumor progression has not been fully elucidated. In the present study, we demonstrated that the proliferation and colony formation ability of CC cells were significantly inhibited after transfection with siMGP. With Fluo-3 AM staining, a direct decrease of free Ca^{2+} concentration in the CC cell cytoplasm was observed. In addition, we observed the phosphorylation levels of three Ca^{2+}-associated transcription factors; NF-κB p65, CREB, and NFATC1 were decreased after blocking the expression level of MGP with siRNA in CC cells. Therefore, we suggested that MGP could affect Ca^{2+}-associated regulating pathways by directly regulating free Ca^{2+} concentration inside cells. Generally, the homeostasis of cytosolic Ca^{2+} was well controlled by calcium ion channels on the plasma membrane and intracellular organelles.[36] It is known that MGP could bind to Ca^{2+} via Gla residues. Thus, MGP located inside CC cells could interfere with the homeostasis of Ca^{2+} by incorporating Ca^{2+} and increasing the intracellular Ca^{2+} level. However, whether MGP-incorporated Ca^{2+} ions have the same activity in regulating the downstream pathways (NF-κB, CREB, and NFAT) as the free Ca^{2+} ions in the cytoplasm still needs further investigation. Herein, we suggested that MGP upregulated intracellular free Ca^{2+} concentration and played a crucial role in modulating downstream cellular signaling pathways. Accordingly, we revealed that siMGP inhibited the proliferation of the CC cells by reducing the intracellular free Ca^{2+} concentration and suppressed Ca^{2+}-related physiological processes.

For a long time, MGP was merely considered as a calcification regulator that decreased abnormally high intracellular free Ca^{2+} concentrations through various molecular mechanisms. On the one hand, MGP functioned as a calcium inhibitor via binding to calcium and inhibiting calcium-phosphate precipitation. On the other hand, extracellular vesicles loaded with MGP and apoptotic bodies rich in calcium were both secreted into the extracellular space to prevent apoptosis in vascular smooth muscle cells.[37] It is also noteworthy that extracellular MGP could also affect CC cells by binding to free Ca^{2+} and decreasing extracellular free Ca^{2+} level. Thus, the mechanisms of how MGP affects the concentration of Ca^{2+} inside and outside of CC cells should be a comprehensive issue.

The NF-κB signaling pathway has been well studied in the regulation of various types of cancer.[38,39] In this study, we showed that MGP could promote NF-κB phosphorylation through upregulating intracellular free Ca^{2+} concentration and activate its downstream gene expression.

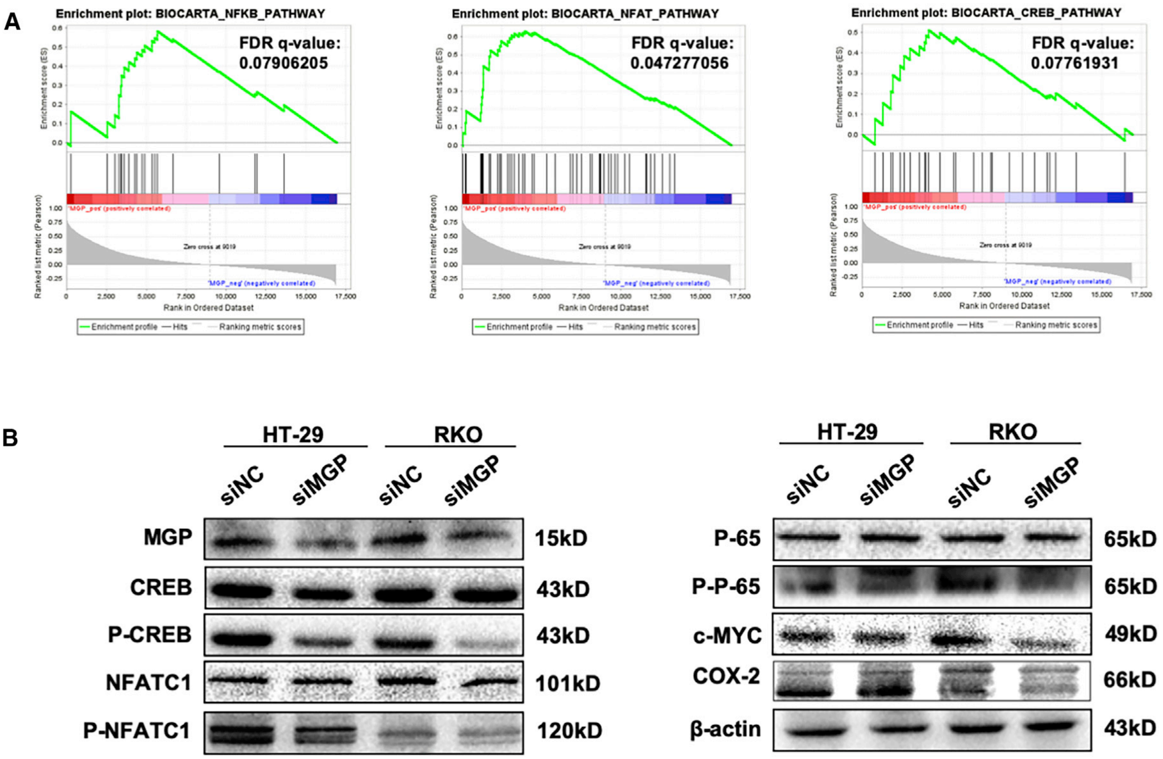

Figure 4. MGP Is Related to Activation of Calcium-Associated Transcription Factors, Including the NF-κB, CREB, and NFAT Signaling Pathways

(A) GSEA indicated that the expression level of MGP was correlated with upregulated NF-κB, CREB, and NFAT pathways. (B) Western blot shows MGP knockdown downregulated the phosphorylation of NF-κB p65, CREB, NFATC1 (left panel), and inhibited NF-κB p65 signaling pathway (right panel).

In previous studies, it was indicated that when the cells were stimulated, the phosphorylated IκBα (inhibitory NF-κBα) was ubiquitinated and degraded, after which the NF-κB heterodimer was translocated into the nucleus and bound to the target gene's κB enhancer site while the target gene was regulated by the transcriptional activation domain (TAD).[40] Through a cytoplasm-nucleus extraction assay and immunofluorescence, we found that the phosphorylated NF-κB level decreased more significantly in the nucleus compared to its change in the cytoplasm. This result indicated that MGP could regulate the mechanism of tumor development by directly or indirectly activating NF-κB. We selected some downstream genes to verify this finding, among which MCL1, BCL2, ICAM-1, and ADH1B were principally related to the anti-apoptotic and proliferative ability of tumors. Quantitative real-time PCR assays verified that ICAM-1 and VEGFA genes were downregulated significantly in both HT-29 and RKO cell lines under siRNA knockdown of MGP. Meanwhile, when overexpressing MGP by lentivirus MGP-Flag, ICAM-1 and VEGFA were evidently increased.

We also found that interference of MGP in normal colonocytes (e.g., CCC-HIE-2) caused the increase of apoptosis cells and the inhibition of cell proliferation and growth, similar to CC cells. We tried several times to transfect lentivirus (MGP-Flag) into CCC-HIE-2 and to sort with different doses of puromycin. However, we could not build a stably transfected MGP-Flag CCC-HIE-2 cell line. It would be possible that excessive MGP was harmful to normal colonocytes, even if it is a growth promoter in cancer cells.

In conclusion, our findings reveal that MGP activates and promotes the phosphorylation of NF-κB by upregulating free calcium concentrations in the cytoplasm. We also highlight the role of cytoplasmic MGP in CC cell growth and proliferation and its potential as a novel biomarker and a target for treatment.

MATERIALS AND METHODS

Patients and Tissue Specimens

A total of 80 pairs of CC and adjacent non-tumor tissues with complete clinicopathologic characteristics were used for IHC staining. All specimens were collected from the paraffin-embedded pathological specimens of patients after surgical resection, who were histopathologically and clinically diagnosed with CC in Beijing Friendship Hospital, Capital Medical University. This study protocol was approved by the Ethics Committee of Beijing Friendship Hospital, Capital Medical University.

IHC

After incubation at 65°C for 1 h, the slides were deparaffinized in xylene and then rehydrated in alcohol. Following antigen removal with high pressure, endogenous peroxidase activity was blocked with

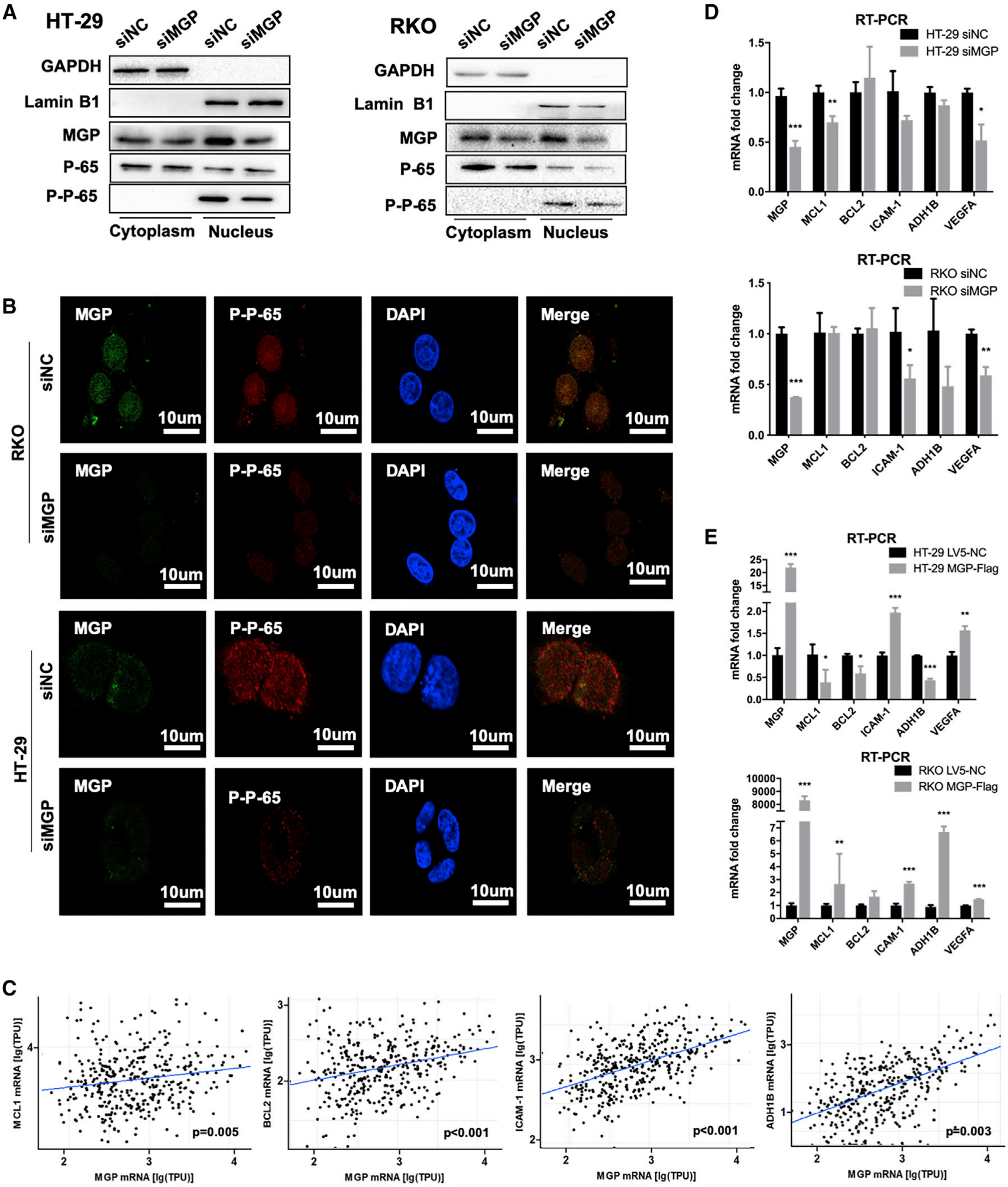

Figure 5. MGP Participates in the Regulation of NF-κB Downstream Molecules

(A) Nucleus and cytoplasm protein separation/extraction assay demonstrates that MGP facilitates NF-κB p65 entering the nucleus. GAPDH was used as a cytoplasm protein marker and lamin B1 served as a nucleus marker. (B) Immunofluorescence assay indicated that NF-κB p65 decreased significantly under MGP siRNA knockdown. DAPI was used to stain nuclei. (C) *In silico* analysis indicates the associations between mRNA expression levels of MGP and the target genes MCL1, BCL2, ICAM-1, and ADH1B (original data were extracted from TCGA CRC dataset). (D) MGP siRNA knockdown decreased the mRNA expression level of NF-κB p65 targeting genes in HT-29 and RKO cells. (E) MGP overexpression increased the mRNA expression level of NF-κB p65 targeting genes in two cell lines. The detected gene expressions of MCL1, BCL2, ICAM-1, ADH1B, and VEGFA were determined by quantitative real-time PCR. Results are representative of three independent experiments. Values are the mean ± SD of the results. *p < 0.05, **p < 0.01, ***p < 0.001.

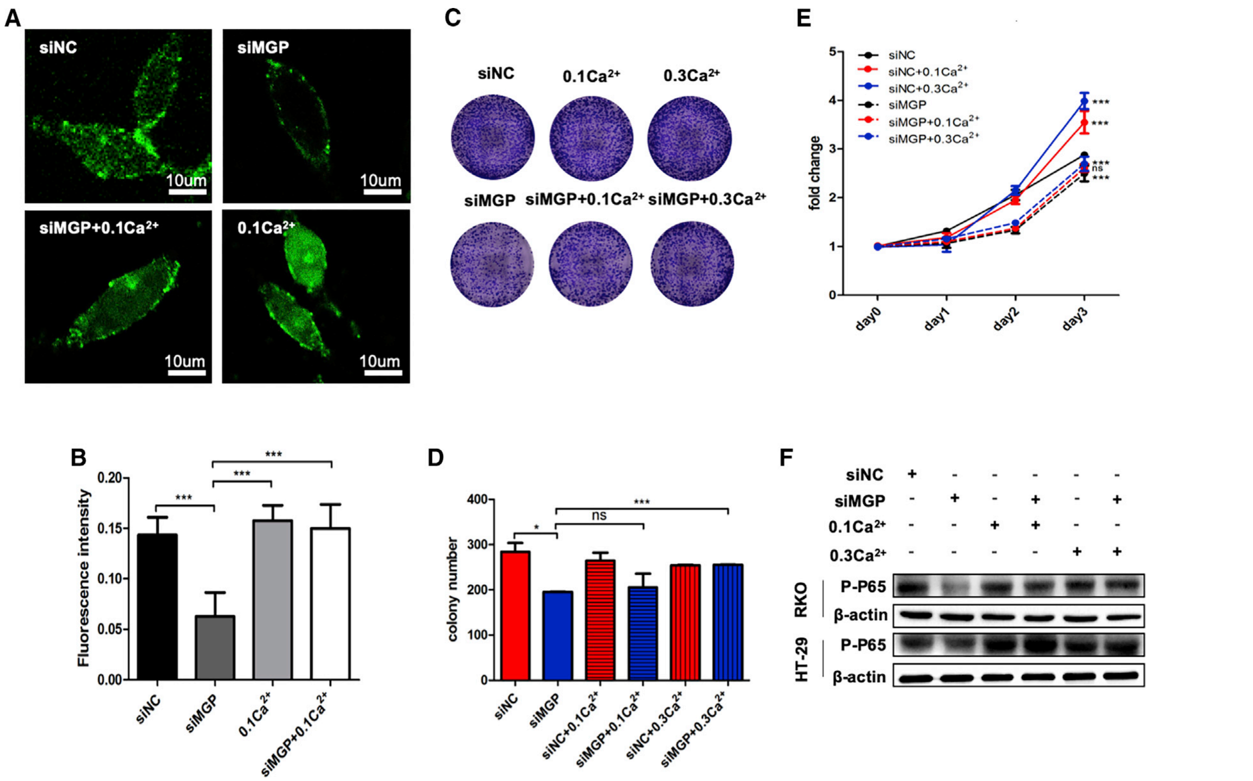

Figure 6. Calcium Is the Essential Mediator for the Pro-proliferation Effect of MGP

(A) After transfection of siMGP into tumor cells for 6–8 h, RKO cells were treated with 0.1 mg/mL calcium concentration medium for 48 h. The living cells were observed under a confocal laser microscope after Fluo-3 AM staining. Random images were taken and the average intracellular fluorescence intensity was calculated. (B) Changes of intracellular fluorescence intensity after treatment with calcium medium. Statistical analysis of three independent experiments is shown. (C and D) Effects of different kinds of Ca^{2+} concentrations on the colon-forming ability of colon cancer cells (C, representative images; D, statistical results of three independ experiments). (E) Effects of different kinds of Ca^{2+} concentrations on the proliferation of colon cancer cells. (F) siMGP-treated RKO and HT-29 cells displayed a decreased expression level of p-NF-κB p65. After treatment with 0.1 and 0.3 mg/mL calcium concentration medium, the expression alteration of p-NF-κB p65 was partially reversed in 48 h. Results are representative of three independent experiments. Values are the mean ± SD of the results. *p < 0.05, **p < 0.01, ***p < 0.001.

3% H_2O_2 for 20–30 min. Sections were blocked by goat serum for 1 h and then incubated at 4°C overnight with the primary antibody against MGP (1:100). After incubation in universal anti-mouse secondary antibody for 2 h at room temperature, tissues were stained using a diaminobenzidine (DAB) kit. Finally, after counterstaining with hematoxylin, IHC slides of normal and tumor tissues were scored based on the staining intensity of the cytoplasm and nucleus, respectively. IHC staining scores were reviewed by two pathologists and divided into two important parts: the staining intensity (0, negative; 1, weakly positive; 2, moderately positive; and 3, strongly positive) and the staining range (0, negative; 1, 1%–33%; 2, 34%–66%; and 3, 67%–100%). The final score was the sum of the two parts of the results.

Cell Culture

Two CC cell lines (HT-29 and RKO) and normal colon epithelial cells (CCC-HIE-2) were purchased from American Type Culture Collection (ATCC). The CC cell lines were cultured in Dulbecco's modified Eagle's medium (DMEM; Gibco, USA), supplemented with 10% fetal bovine serum (FBS; Gibco, USA) at 37°C in an incubator with 5% CO_2. The normal colon epithelial cell was cultured in DMEM, supplemented with 10% FBS (Gibco, Australia). Cell lines used in the experiments were performed for fewer than five passages.

Transfection Reagents

HT-29 and RKO cells were seeded into six-well plates until 50% confluence. MGP siRNAs were synthesized in Suzhou GenePharma and transfected into two cancer cell lines by using Lipofectamine 3000. The transfection efficacy was confirmed by western blot and quantitative real-time PCR. Target sequences of siMGP utilized in the study are listed as follows: forward, 5′-GAUAAGUAAUGAAA GUGCATT-3′; reverse, 5′-UGCACUUUCAUUACUUAUCTT-3′. Also, a nonsilencing NC sequence was as follows: forward, 5′-UUC UUCGAACGUGUCACGUTT-3′; reverse, 5′-ACGUGACACGUU CGGAGAATT-3′. For the overexpression of MGP, the full-length open reading frame (ORF) of MGP was cloned into LV5 and then packaged in lentivirus with a 3× Flag-tag and the eukaryotic resistance gene of purine (Suzhou Gene Pharma). Stable transfections of the gene MGP were selected for at least 1 week with puromycin.

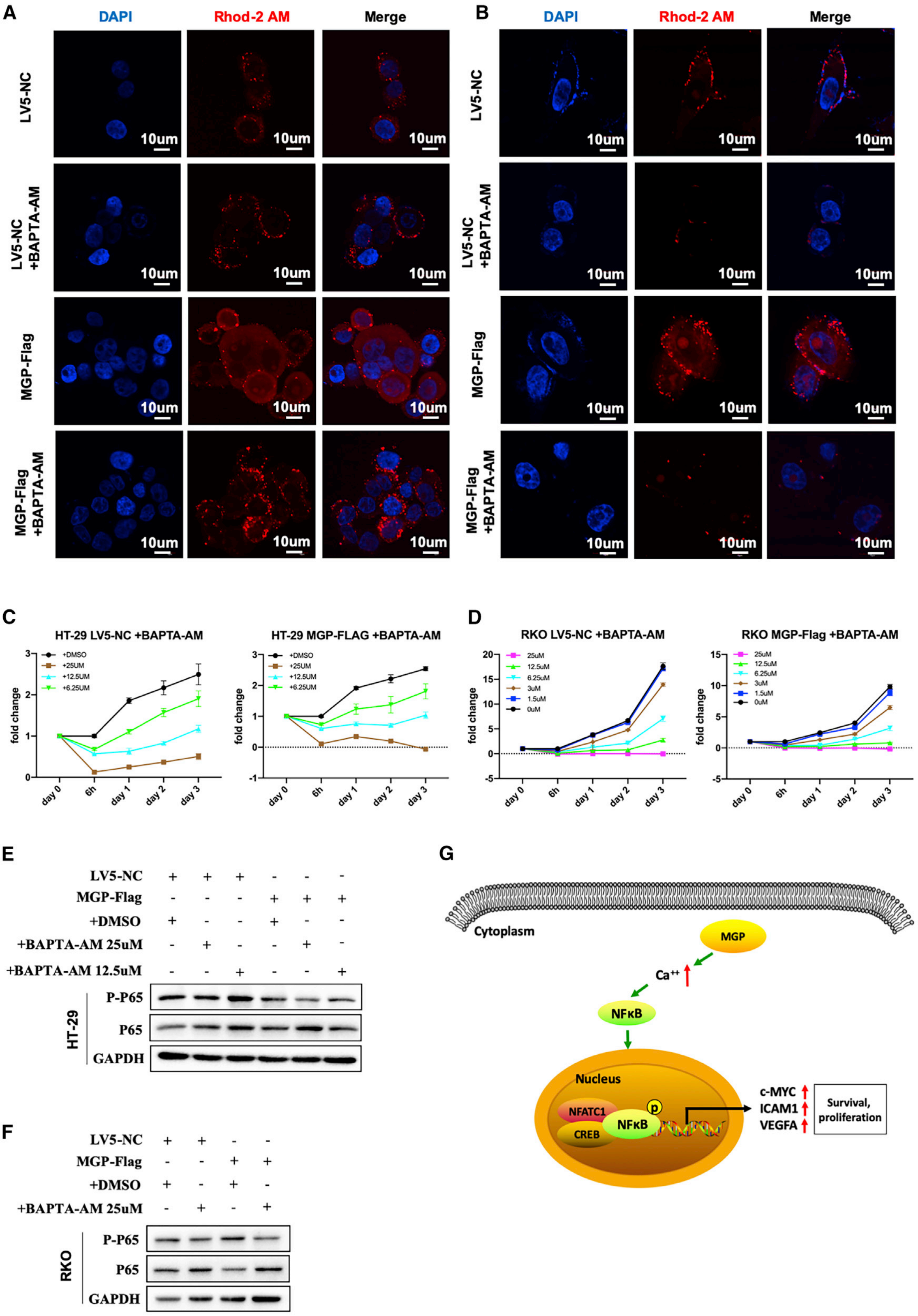

(legend on next page)

Cell Viability Assay and Colony Formation Assay

To explore the functional role of MGP on the proliferation of cells, one-step MTS assays were performed. A total of 3,000 cells/well in 100 μL of medium were seeded in a 96-well plate after transfection, and 20 μL of MTS reagent/well was added at the time points of 0, 24, 48, and 72 h. After incubating at 37°C for 2 h, an enzyme-labeled meter (SpectraMax M3, Molecular Devices) was used to access the cell viability. For the colony formation assay, 1,000 cells of two cell lines were plated in six-well plates after transfection. Subsequently, cells were fixed and stained with 0.1% crystal violet when visible colonies were formed during the following 10–15 days. Three independent experiments were performed for all assays.

Cell Apoptosis Detection Assay

After HT-29, RKO, and CCC-HIE-2 cell lines were transfected with siRNA for 48 h, cells were digested and washed twice in Dulbecco's phosphate-buffered saline (DPBS), then resuspended in 1× binding buffer. An annexin V-FITC (fluorescein isothiocyanate)/7-AAD (7-aminoactinomycin D) staining kit (BD Biosciences, San Jose, CA, USA) was utilized for cell staining. The apoptosis rate was detected by FACS software after incubation with the staining kit for 15 min according to the manufacturer's protocol.

EdU Incorporation Assay

For EdU assays (RiboBio, China), cells were attached to the 24-well plate for 24 h and then cultured with 10 nM EdU solution at 37°C for 2 h, followed by fixation in 4% formaldehyde for 30 min. Then, the cells were treated with an Apollo cocktail for 50 min and subsequently treated with Hoechst 33342 for 40 min for nuclear staining. Finally, cell proliferation was detected under an inverted fluorescence microscope (Axio Observer Z1, Carl Zeiss, Germany). Three independent experiments were performed for all assays.

Fluo-3 AM Staining

The stock solution (5 mM) of Fluo-3 AM (Biyuntian, China) was diluted with Hanks' balanced salt solution (HBSS) (0.39 g/L KCl, 0.07 g/L KH_2PO_4, 8.06 g/L NaCl, 0.10 g/L $Na_2HPO_4 \cdot 7H_2O$, 0.24 g/L $CaCl_2$, 0.10 g/L $MgCl_2$, 0.10 g/L $MgSO_4$, and 1.52 g/L D-glucose) to give the final concentrations of 5 μM. The entire staining process of Fluo-3 AM involves two stages: loading and de-esterification. The investigated cells were loaded with 5 μM Fluo-3 AM at a loading temperature (TL) of 37°C. Then, they were washed in pure HBSS and left for a further 30 min at a de-esterification temperature (TD) of 37°C to allow de-esterification. Cells were then imaged by confocal microscopy (IX83, FluoView FV1200, Olympus). We randomly selected the field of view to take images and calculated the average intracellular fluorescence intensity for statistics.

After loading with Fluo-3 AM, CC cells were digested with trypsin and washed with HBSS three times, after which we measured the intracellular calcium ion concentration curves by FACS software. Three independent experiments were performed for all assays.

Rhod-2 AM Staining

Intracellular calcium levels of CC cells stably transfected with lentivirus were analyzed using a calcium kit (Rhod-2 AM, Abcam, USA) according to the manufacturer's protocol. Cells were then imaged by confocal microscopy (IX83, FluoView FV1200, Olympus).

Western Blot Analysis

After protein quantification with a bicinchoninic acid (BCA) protein assay kit (Thermo Fisher Scientific), a total of 50 μg of denatured proteins per line underwent electrophoresis. Then, the proteins were transferred to polyvinylidene fluoride (PVDF) membranes and blocked by 5% (w/v) milk (non-fat milk in Tris-buffered saline with Tween 20 [TBST]). Membranes were incubated in primary antibodies against MGP, NF-κB p65, p-NF-κB p65, CREB, p-CREB, NFATC1, p-NFATC1, β-actin, GAPDH, c-MYC, and COX-2 at 4°C overnight. Antibodies used in the study are listed in Table S3. The following day, after washing with TBST three times, membranes were incubated in peroxidase-conjugated secondary antibodies for 1 h at room temperature. After washing with TBST for another six times, the detection of protein bands was performed with an enhanced chemiluminescence system (Bio-Rad, USA).

Nucleus and Cytoplasm Extraction Assay

Nucleus and cytoplasm extraction reagents (Thermo Scientific, 78833) were applied in this experiment. The operational procedures were all performed in strict accordance with the protocol provided by the manufacturer. Western blot assays were carried out to detect the protein; GAPDH acted as the marker of cytoplasm protein and lamin B1 as the marker of nucleus protein.

Immunofluorescence (IF)

To evaluate the cellular localization of MGP and NF-κB p65, HT-29 and RKO cell lines were seeded on sterile coverslips in six-well plates until 50% confluence, washed with PBS three times, and fixed in 4% paraformaldehyde for 15 min. After blocking in 5% BSA in PBST for 1 h, cells were permeabilized with 0.25% Triton X-100 in PBS. Anti-MGP (1:50), anti-NF-κB p65 (1:50), and anti-p-NF-κB p65 (1:50) were mixed in PBS and used for sample incubation overnight at 4°C. Cells were subsequently incubated in a mixture of two kinds of fluorescent secondary antibodies (Alexa Fluor 488-conjugated anti-mouse immunoglobulin G [IgG; 1:200] and Alexa Fluor 594-conjugated anti-rabbit IgG [1:200]) (Life Technologies) in the dark

Figure 7. MGP-Induced Pro-Proliferation Ability Is Blocked by Sequestering the Intracellular Calcium Ion in CC Cells

(A and B) HT-29 (A) and RKO (B) cell lines stably transfected with lentivirus vector (MGP-Flag) were preincubated for 4 h with BAPTA-AM (25 μM). The concentrations of Ca^{2+} in CC cells were observed under a confocal laser microscope stained with Rhod-2 AM (red) and DAPI (blue). (C and D) Different concentrations of BAPTA-AM were cocultured with CC cells, and the distinct effects on cell proliferation were evaluated by an MTS assay (C, HT-29 cells; D, RKO cells). (E and F) After treatment with BAPTA-AM (25 μM) for 4 h, the expression alteration of p-NF-κB p65 was decreased, especially in the RKO cell line (E, HT-29 cells; F, RKO cells). (G) The simplified diagram shows the mechanism by which MGP promotes CC growth and proliferation through upregulating intracellular free calcium concentration and activating the NF-κB signaling pathway.

for 2 h and then stained with DAPI and imaged by confocal microscopy (IX83, FluoView FV1200, Olympus).

RNA Extraction and Quantitative Real-Time PCR

Total RNA was extracted using TRIzol (Invitrogen, Karlsruhe, Germany) from two cell lines. Quantitative real-time PCR was performed using a SYBR Green mix (Invitrogen) and run in an ABI 7500 real-time PCR system (Applied Biosystems) with cycling parameters listed as follows: 94°C for 2 min followed by 40 cycles of 94°C for 15 s, 56°C for 20 s, and 72°C for 30 s and then followed by 72°C for 2 min. With a melting curve analysis, ΔΔCT was used to calculate the relative gene expression of qPCR. Primers detected in the study are as follows: GAPDH forward, 5′-GGAGCGAGATCCCTCCAAAAT-3′, reverse, 5′-GGCTGTTGTCATACTTCTCATGG-3′; MCL1 forward, 5′-GGCCTTCCAAGGATGGGTTT-3′, reverse, 5′-ACTCCAGCAACACCTGCAAAA-3′; BCL2 forward, 5′-GGTGGGGTCATGTGTGTGG-3′, reverse, 5′-CGGTTCAGGTACVTCAGTCATCC-3′; ICAM-1 forward, 5′-ATGCCCAGACATCTGTGTCC-3′, reverse, 5′-GGGGTCTCTATGCCCAACAA-3′; ADH1B forward, 5′-ATTGGCTGTGGATTCTCGAC-3′, reverse 5′-ATTCAGTGGCACCCAACTCT-3′; VEGFA forward, 5′-TGTGTGGGTGAGTGAGTGTG-3′, reverse, 5′-TATTGGAATCCTGGAGTGACC-3′. Three independent experiments were performed for all assays.

Statistical Analysis

Data represent mean ± SD; all statistical data were analyzed using GraphPad Prism 5 and R software. Unpaired, two-sided Student's t tests were conducted to compare the differences between two groups. Kaplan-Meier plots and log rank tests were applied to assess and show the difference in overall survival (OS) and disease-free survival (DFS) between subgroups. Cox models were used for multiple variants analysis. $p < 0.05$ was considered to indicate a statistically significant difference.

Data Accessibility

The datasets and materials used for the study are available from the corresponding author on reasonable request.

SUPPLEMENTAL INFORMATION

Supplemental Information can be found online at https://doi.org/10.1016/j.omto.2020.04.005.

AUTHOR CONTRIBUTIONS

L. Min and S. Zhang conceived and designed the study. X.L., R.W., M.W. and L. Ma performed all experiments. Z.Z., L.C., Q.G., and S. Zhu helped to collect, reformat, and analyze the primary data. R.W. and L. Min drafted the manuscript. X.L., S.G., L. Min, and S. Zhang proofread and revised the manuscript. All authors read and approved the final manuscript.

CONFLICTS OF INTEREST

The authors declare no competing interests.

ACKNOWLEDGMENTS

We thank Dr. Guangyong Chen and Rui Xu, Department of Pathology, Beijing Friendship Hospital, for the supply of tissue materials and the score assessment for immunohistochemistry staining. We thank Yun Zhang and the Clinical Data and Biobank Resource of Beijing Friendship Hospital for help in sample collection. We appreciate the help from all colleagues. This work was fully supported by National Natural Science Foundation of China (81702314), Funding Program for Excellent Talents of Beijing (2017000021469G212), Beijing Municipal Administration of Hospitals' Youth Program (QML20180108), and by the Digestive Medical Coordinated Development Center of Beijing Municipal Administration of Hospitals (XXZ0201).

REFERENCES

1. Bray, F., Ferlay, J., Soerjomataram, I., Siegel, R.L., Torre, L.A., and Jemal, A. (2018). Global cancer statistics 2018: GLOBOCAN estimates of incidence and mortality worldwide for 36 cancers in 185 countries. CA Cancer J. Clin. *68*, 394–424.
2. Papadopoulos, V., Tsapakidis, K., Riobo Del Galdo, N.A., Papandreou, C.N., Del Galdo, F., Anthoney, A., Sakellaridis, N., Dimas, K., and Kamposioras, K. (2016). The prognostic significance of the hedgehog signaling pathway in colorectal cancer. Clin. Colorectal Cancer *15*, 116–127.
3. Siegel, R., Desantis, C., and Jemal, A. (2014). Colorectal cancer statistics, 2014. CA Cancer J. Clin. *64*, 104–117.
4. De Roock, W., De Vriendt, V., Normanno, N., Ciardiello, F., and Tejpar, S. (2011). *KRAS*, *BRAF*, *PIK3CA*, and *PTEN* mutations: implications for targeted therapies in metastatic colorectal cancer. Lancet Oncol. *12*, 594–603.
5. Wang, B., Shen, Z.L., Jiang, K.W., Zhao, G., Wang, C.Y., Yan, Y.C., Yang, Y., Zhang, J.Z., Shen, C., Gao, Z.D., et al. (2015). MicroRNA-217 functions as a prognosis predictor and inhibits colorectal cancer cell proliferation and invasion via an AEG-1 dependent mechanism. BMC Cancer *15*, 437.
6. Price, P.A., Urist, M.R., and Otawara, Y. (1983). Matrix Gla protein, a new γ-carboxyglutamic acid-containing protein which is associated with the organic matrix of bone. Biochem. Biophys. Res. Commun. *117*, 765–771.
7. Price, P.A., and Williamson, M.K. (1985). Primary structure of bovine matrix Gla protein, a new vitamin K-dependent bone protein. J. Biol. Chem. *260*, 14971–14975.
8. Fraser, J.D., and Price, P.A. (1988). Lung, heart, and kidney express high levels of mRNA for the vitamin K-dependent matrix Gla protein. Implications for the possible functions of matrix Gla protein and for the tissue distribution of the gamma-carboxylase. J. Biol. Chem. *263*, 11033–11036.
9. Shanahan, C.M., Cary, N.R., Metcalfe, J.C., and Weissberg, P.L. (1994). High expression of genes for calcification-regulating proteins in human atherosclerotic plaques. J. Clin. Invest. *93*, 2393–2402.
10. Ketteler, M., Vermeer, C., Wanner, C., Westenfeld, R., Jahnen-Dechent, W., and Floege, J. (2002). Novel insights into uremic vascular calcification: role of matrix Gla protein and alpha-2-Heremans Schmid glycoprotein/fetuin. Blood Purif. *20*, 473–476.
11. Theuwissen, E., Smit, E., and Vermeer, C. (2012). The role of vitamin K in soft-tissue calcification. Adv. Nutr. *3*, 166–173.
12. Munroe, P.B., Olgunturk, R.O., Fryns, J.P., Van Maldergem, L., Ziereisen, F., Yuksel, B., Gardiner, R.M., and Chung, E. (1999). Mutations in the gene encoding the human matrix Gla protein cause Keutel syndrome. Nat. Genet. *21*, 142–144.
13. Sun, L.-F., and Chen, X. (2012). Tracheobronchial stenosis in Keutel syndrome. Indian Pediatr. *49*, 759.
14. Mertsch, S., Schurgers, L.J., Weber, K., Paulus, W., and Senner, V. (2009). Matrix gla protein (MGP): an overexpressed and migration-promoting mesenchymal component in glioblastoma. BMC Cancer *9*, 302.
15. Hackeng, T.M., Rosing, J., Spronk, H.M., and Vermeer, C. (2001). Total chemical synthesis of human matrix Gla protein. Protein Sci. *10*, 864–870.

16. Wallin, R., Cain, D., Hutson, S.M., Sane, D.C., and Loeser, R. (2000). Modulation of the binding of matrix Gla protein (MGP) to bone morphogenetic protein-2 (BMP-2). Thromb. Haemost. *84*, 1039–1044.

17. Hale, J.E., Fraser, J.D., and Price, P.A. (1988). The identification of matrix Gla protein in cartilage. J. Biol. Chem. *263*, 5820–5824.

18. Luo, G., Ducy, P., McKee, M.D., Pinero, G.J., Loyer, E., Behringer, R.R., and Karsenty, G. (1997). Spontaneous calcification of arteries and cartilage in mice lacking matrix GLA protein. Nature *386*, 78–81.

19. Yao, Y., Shahbazian, A., and Boström, K.I. (2008). Proline and γ-carboxylated glutamate residues in matrix Gla protein are critical for binding of bone morphogenetic protein-4. Circ. Res. *102*, 1065–1074.

20. Rasmussen, H., Barrett, P., Smallwood, J., Bollag, W., and Isales, C. (1990). Calcium ion as intracellular messenger and cellular toxin. Environ. Health Perspect. *84*, 17–25.

21. Zheng, J., Zeng, X., and Wang, S. (2015). Calcium ion as cellular messenger. Sci. China Life Sci. *58*, 1–5.

22. Stewart, T.A., Yapa, K.T.D.S., and Monteith, G.R. (2015). Altered calcium signaling in cancer cells. Biochim. Biophys. Acta *1848* (10 Pt B), 2502–2511.

23. Krebs, J. (1998). The role of calcium in apoptosis. Biometals *11*, 375–382.

24. Taylor, J.T., Zeng, X.-B., Pottle, J.E., Lee, K., Wang, A.R., Yi, S.G., Scruggs, J.A., Sikka, S.S., and Li, M. (2008). Calcium signaling and T-type calcium channels in cancer cell cycling. World J. Gastroenterol. *14*, 4984–4991.

25. Schmidt, A., Oberle, N., Weiss, E.-M., Vobis, D., Frischbutter, S., Baumgrass, R., Falk, C.S., Haag, M., Brügger, B., Lin, H., et al. (2011). Human regulatory T cells rapidly suppress T cell receptor-induced Ca^{2+}, NF-κB, and NFAT signaling in conventional T cells. Sci. Signal. *4*, ra90.

26. Wang, Y., Deng, X., Mancarella, S., Hendron, E., Eguchi, S., Soboloff, J., Tang, X.D., and Gill, D.L. (2010). The calcium store sensor, STIM1, reciprocally controls Orai and CaV1.2 channels. Science *330*, 105–109.

27. Houben, E., Neradova, A., Schurgers, L.J., and Vervloet, M. (2016). The influence of phosphate, calcium and magnesium on matrix Gla-protein and vascular calcification: a systematic review. G. Ital. Nefrol. *33*, 10.

28. Biswas, G., Guha, M., and Avadhani, N.G. (2005). Mitochondria-to-nucleus stress signaling in mammalian cells: nature of nuclear gene targets, transcription regulation, and induced resistance to apoptosis. Gene *354*, 132–139.

29. Gheorghe, S.R., and Crăciun, A.M. (2016). Matrix Gla protein in tumoral pathology. Clujul Med. *89*, 319–321.

30. Fan, C., Sheu, D., Fan, H., Hsu, K., Allen Chang, C., and Chan, E. (2001). Down-regulation of matrix Gla protein messenger RNA in human colorectal adenocarcinomas. Cancer Lett. *165*, 63–69.

31. Micke, P., Kappert, K., Ohshima, M., Sundquist, C., Scheidl, S., Lindahl, P., Heldin, C.H., Botling, J., Ponten, F., and Ostman, A. (2007). In situ identification of genes regulated specifically in fibroblasts of human basal cell carcinoma. J. Invest. Dermatol. *127*, 1516–1523.

32. Clapham, D.E. (2007). Calcium signaling. Cell *131*, 1047–1058.

33. Gilon, P., Chae, H.-Y., Rutter, G.A., and Ravier, M.A. (2014). Calcium signaling in pancreatic β-cells in health and in type 2 diabetes. Cell Calcium *56*, 340–361.

34. Romero-Garcia, S., and Prado-Garcia, H. (2019). Mitochondrial calcium: transport and modulation of cellular processes in homeostasis and cancer (Review). Int. J. Oncol. *54*, 1155–1167.

35. Fourbon, Y., Guéguinou, M., Félix, R., Constantin, B., Uguen, A., Fromont, G., Lajoie, L., Magaud, C., Lecomte, T., Chamorey, E., et al. (2017). Ca^{2+} protein alpha 1D of CaV1.3 regulates intracellular calcium concentration and migration of colon cancer cells through a non-canonical activity. Sci. Rep. *7*, 14199.

36. Zampese, E., and Pizzo, P. (2012). Intracellular organelles in the saga of Ca^{2+} homeostasis: different molecules for different purposes? Cell. Mol. Life Sci. *69*, 1077–1104.

37. Schurgers, L.J., Uitto, J., and Reutelingsperger, C.P. (2013). Vitamin K-dependent carboxylation of matrix Gla-protein: a crucial switch to control ectopic mineralization. Trends Mol. Med. *19*, 217–226.

38. Dolcet, X., Llobet, D., Pallares, J., and Matias-Guiu, X. (2005). NF-kB in development and progression of human cancer. Virchows Arch. *446*, 475–482.

39. Hassanzadeh, P. (2011). Colorectal cancer and NF-κB signaling pathway. Gastroenterol. Hepatol. Bed Bench *4*, 127–132.

40. Christian, F., Smith, E.L., and Carmody, R.J. (2016). The regulation of NF-κB subunits by Phosphorylation. Cells *5*, 12.

OMTO, Volume *17*

Supplemental Information

MGP Promotes Colon Cancer Proliferation by Activating the NF-κB Pathway through Upregulation of the Calcium Signaling Pathway

Xueqing Li, Rui Wei, Mizhu Wang, Li Ma, Zheng Zhang, Lei Chen, Qingdong Guo, Shuilong Guo, Shengtao Zhu, Shutian Zhang, and Li Min

A

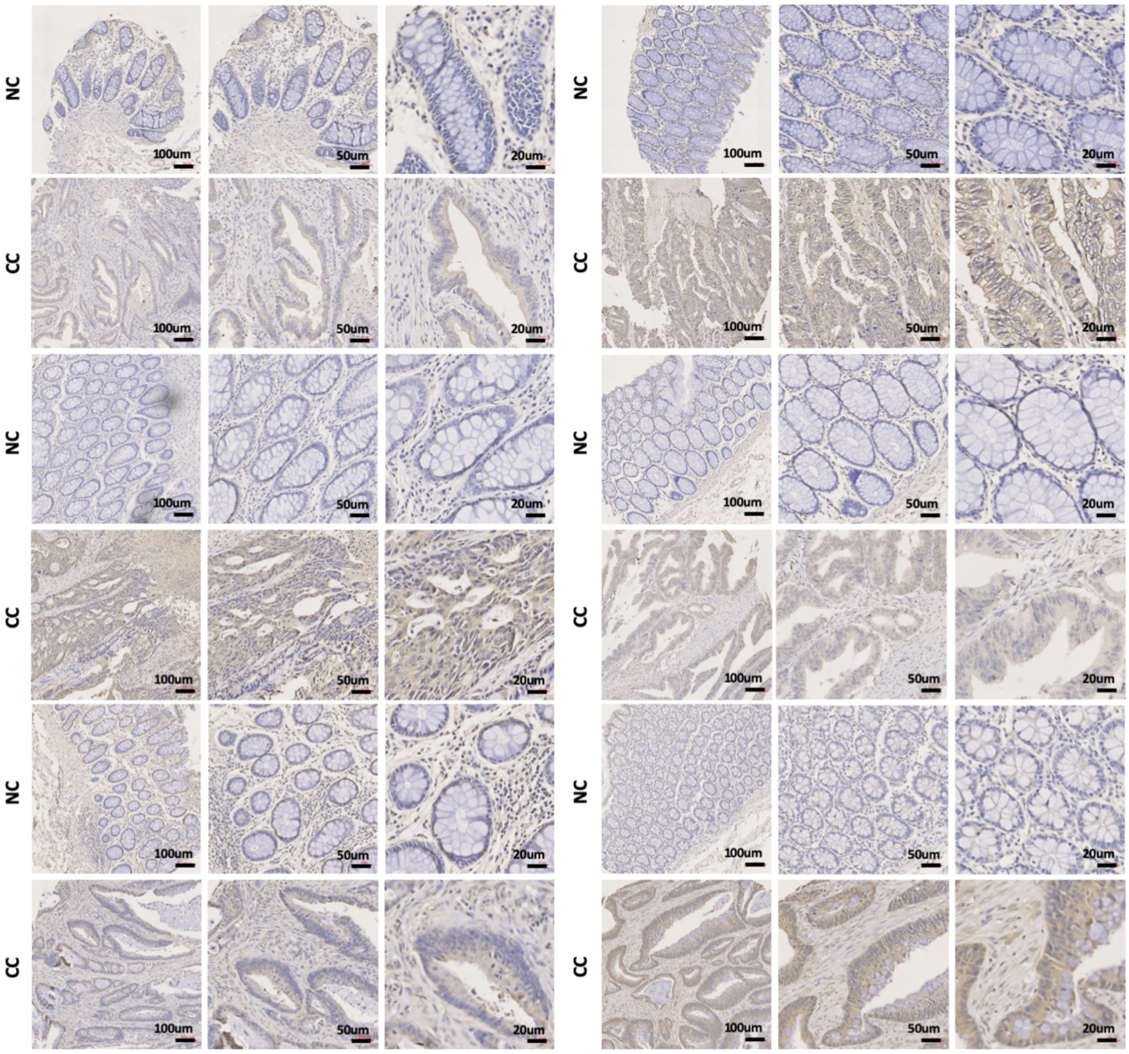

Figure S1. MGP is up-regulated in colon cancer patients.

(A) Representative IHC pictures of 80 pairs of CC patients. NC, adjacent non-tumor tissues; CC, colon cancer tissues.

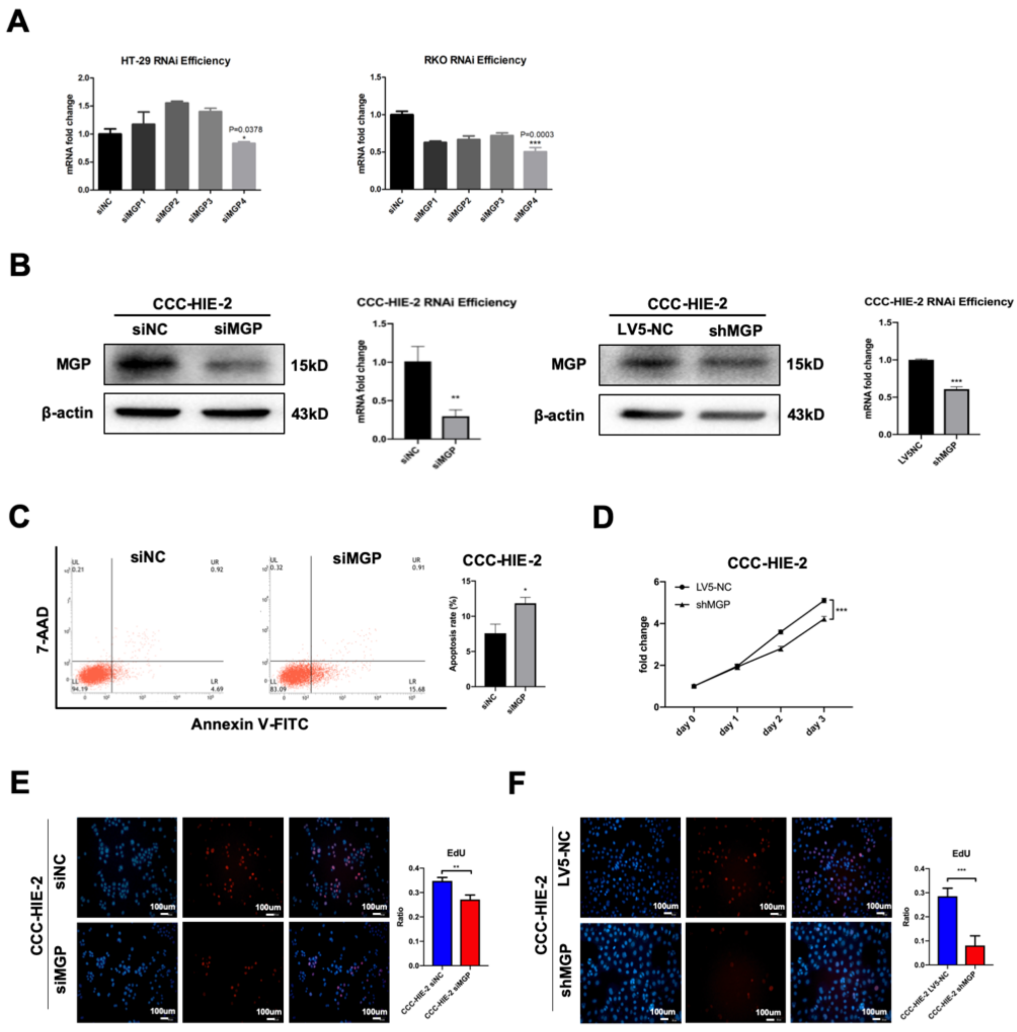

Figure S2. MGP inhibits cell apoptosis and promotes cell proliferation in normal colon epithelial cell.

(A) We designed four kinds of siRNA, and verified the knockdown efficiency by RT-qPCR after transfected with siRNAs in HT-29 and RKO cell lines. (B) The knockdown efficiency of normal colon epithelial cell line, CCC-HIE-2, was identified by western blotting and qRT-PCR. (C) The apoptosis ability of CCC-HIE-2 after transfected with siMGP. (D) The growth curve of CCC-HIE-2 after knockdown MGP. (E) EdU staining of CCC-HIE-2 transfected with siMGP. (F) EdU staining of CCC-HIE-2 stably transfected with LV5-NC and shMGP. Results are representative of 3 independent experiments. Values are the mean ±SD of the results. *p <0.05; **p <0.01; ***p <0.001.

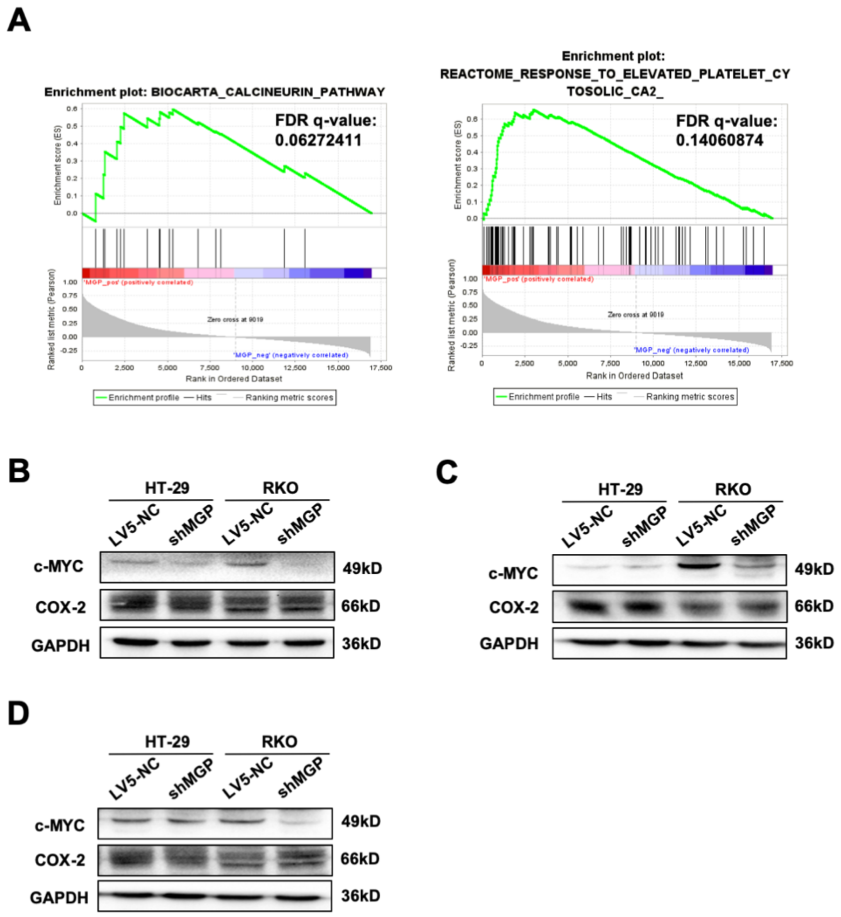

Figure S3. MGP is associated with calcium related pathways, and regulates NF-κB downstream genes expression in CC cells.

(A) GSEA analysis indicated that the expression level of MGP was correlated with up-regulated calcineurin pathway and elevated platelet cytosolic. (B, C, D) The protein level of NF-κB downstream genes when knockdown MGP.

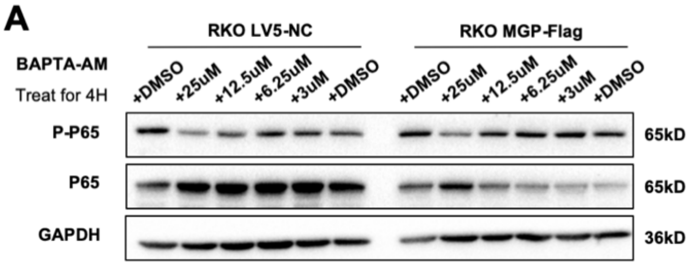

Figure S4. MGP regulating NF-κB pathway is blocked by BAPTA-AM in CC cells.

(A) Different concentrations of BAPTA-AM were cocultured with RKO cells for 4 hours. The protein level of p-NF-κB P65 was decreased at 25uM, with an increase expression of NF-κB P65.

Table S1. Clinical and pathological characteristics of 80 pairs of CC patients.

Variables	No. of patient
**Gender**	
Male	47 (58.75%)
Female	33 (41.25%)
**Age (years)**	
≥ 65	37 (46.25%)
< 65	43 (53.75%)
**T stage**	
T1, T2	15 (18.75%)
T3, T4	61 (76.25%)
*4 pairs of patients' information are not clear.	
**Lymph Node Metastasis**	
Negative	42 (52.50%)
Positive	38 (47.50%)
**Distant Metastases**	
Negative	69 (86.25%)
Positive	11 (13.75%)
**Pathological Stage**	
I-II, II	61 (76.25%)
II-III, III	19 (23.75%)
**Clinical Stage**	
I, II	39 (48.75%)
III, IV	41 (51.25%)

Table S2. The detailed histology scores of 80 pairs of colon cancer patients' tissues. (Excel)

Table S3. Information of primary antibodies in WB and IF.

Primary Antibody	Company	Cat.	Dilution Factor
β-actin	Abbkine	A01010	WB 1:5000
MGP	Santa	SC-81546	IHC 1:100
			WB 1:500
			IF 1:50
NF-κB P65	Proteintech	10745-1-AP	WB 1:2000
			IF 1:50
phospho-NF-κB P65	HuaBio	ET1604-27	WB 1:1000
			IF 1:50
CREB	CST	9197	WB 1:1000
phospho-CREB	CST	9198	WB 1:1000
NFATC1	Abcam	Ab177464	WB 1:1000
phospho-NFATC1	R&D	679340	WB 1:1000
c-MYC	Proteintech	10828-1-AP	WB 1:2000
COX-2	Proteintech	12375-1-AP	WB 1:2000
GAPDH	Abbkine	A01020	WB 1:5000
Lamin B1	CST	9087S	WB 1:1000

Table S4. Details and the raw data of the GSEA (KEGG) analysis for MGP. (Excel)

Table S5. Details and the raw data of the GSEA (BIOCARTA) analysis for MGP. (Excel)
